# cAMP-Epac1 signaling is activated in DDAVP-induced endolymphatic hydrops of guinea pigs

**DOI:** 10.1016/j.bjorl.2023.03.002

**Published:** 2023-03-13

**Authors:** Wang Chuan, Li Yuan, Jiang Wen, Zeng Jianwei, Wang Caiji, Zhao Zeqi, Li Yalan, Ji Renlong, Li Kang, Li Wei, Liu Houguang, Liu Wen, Qiao Yuehua, Li Xuanyi

**Affiliations:** a The Suqian Clinical College of Xuzhou Medical University, Department of Otorhinolaryngology-Head and Neck Surgery, Suqian, China; b Affiliated Hospital of Xuzhou Medical University, Department of Otorhinolaryngology-Head and Neck Surgery, Xuzhou, China; c Xuzhou Medical University, Institute of Audiology and Balance Science, Xuzhou, China; d Xuzhou Medical University, Artificial Auditory Laboratory of Jiangsu Province, Xuzhou, China; e Affiliated Hospital of Xuzhou Medical University, Department of Radiology, Xuzhou, China; f Gulou Hospital Affiliated to Medical College of Nanjing University, Department of Otolaryngology Head and Neck Surgery, Nanjing, China; g Fudan University, Hearing Research Key Lab of Health Ministry of China, Eye and Ear Nose and Throat Hospital, Department of Otology and Skull Base Surgery, Shanghai, China; h China University of Mining and Technology, School of Mechatronic Engineering, Xuzhou, China; i Xuzhou Medical University, Jiangsu Key Laboratory of New Drug Research and Clinical Pharmacy, Xuzhou, China

**Keywords:** Exchange protein directly activated by cAMP (Epac1), Epac2, cAMP-Epac1, Inner ear, Endolymphatic Hydrops (EH), 1-desamino-8-D-arginine-vasopressin (desmopressin acetate, DDAVP)

## Abstract

**Objective:**

To explore whether Cyclic Adenosine Monophosphate (cAMP)-Epac1 signaling is activated in 1-Desamino-8-D-arginine-Vasopressin-induced Endolymphatic Hydrops (DDAVP-induced EH) and to provide new insight for further in-depth study of DDAVP-induced EH.

**Methods:**

Eighteen healthy, red-eyed guinea pigs (36 ears) weighing 200–350 g were randomly divided into three groups: the control group, which received intraperitoneal injection of sterile saline (same volume as that in the other two groups) for 7 consecutive days; the DDAVP-7d group, which received intraperitoneal injection of 10 mg/mL/kg DDAVP for 7 consecutive days; and the DDAVP-14d group, which received intraperitoneal injection of 10 μg/mL/kg DDAVP for 14 consecutive days. After successful modeling, all animals were sacrificed, and cochlea tissues were collected to detect the mRNA and protein expression of the exchange protein directly activated by cAMP-1 and 2 (Epac1, Epac2), and Repressor Activator Protein-1 (Rap1) by Reverse Transcription (RT)-PCR and western blotting, respectively.

**Results:**

Compared to the control group, the relative mRNA expression of Epac1, Epac2, Rap1A, and Rap1B in the cochlea tissue of the DDAVP-7d group was significantly higher (*p* <  0.05), while no significant difference in Rap1 GTPase activating protein (Rap1gap) mRNA expression was found between the two groups. The relative mRNA expression of Epac1, Rap1A, Rap1B, and Rap1gap in the cochlea tissue of the DDAVP-14d group was significantly higher than that of the control group (*p* <  0.05), while no significant difference in Epac2 mRNA expression was found between the DDAVP-14d and control groups. Comparison between the DDAVP-14d and DDAVP-7d groups showed that the DDAVP-14d group had significantly lower Epac2 and Rap1A (*p* <  0.05) and higher Rap1gap (*p* < 0.05) mRNA expression in the cochlea tissue than that of the DDAVP-7d group, while no significant differences in Epac1 and Rap1B mRNA expression were found between the two groups. Western blotting showed that Epac1 protein expression in the cochlea tissue was the highest in the DDAVP-14d group, followed by that in the DDAVP-7d group, and was the lowest in the control group, showing significant differences between groups (*p* <  0.05); Rap1 protein expression in the cochlea tissue was the highest in the DDAVP-7d group, followed by the DDAVP-14d group, and was the lowest in the control group, showing significant differences between groups (*p* <  0.05); no significant differences in Epac2 protein expression in the cochlea tissue were found among the three groups.

**Conclusion:**

DDAVP upregulated Epac1 protein expression in the guinea pig cochlea, leading to activation of the inner ear cAMP-Epac1 signaling pathway. This may be an important mechanism by which DDAVP regulates endolymphatic metabolism to induce EH and affect inner ear function.

**Oxford Centre for Evidence-Based Medicine 2011 Levels of Evidence:**

Level 5.

## Highlights

• This work aims to explore whether cAMP-Epac1 signaling is also activated.
• We verified the differential expression of Epac1 and Epac2 in the inner ear of guinea pigs with endolymphatic hydrops.
• The results of this study showed DDAVP upregulated Epac1 protein expression in the guinea pig cochlea.
• The article laid a theoretical foundation for further investigation on how DDAVP regulates endolymphatic metabolism to induce EH and affect inner ear function.

## Highlights:

- DDAVP upregulated Epac1 protein expression in the guinea pig cochlea,
- It activates the inner ear cAMP-Epac1-Rap1 signaling pathway,
- DDAVP regulates endolymphatic metabolism to induce EH and affect inner ear function.

## Introduction

Meniere’s Disease (MD) is an inner ear disease with unknown etiology and pathogenesis, the pathological feature of which is mainly characterized by Endolymphatic Hydrops (EH). The clinical symptoms of MD mainly include episodic vertigo, fluctuating sensorineural hearing loss, tinnitus, and/or ear fullness^1^, ^2^. EH is caused by excessive endolymph production or absorption obstacles due to various reasons, which results in an increase in lymphatic fluid in the membranous labyrinth. EH is a common pathological change in many ear diseases, including MD, delayed EH, and deafness caused by virus infection^3^, ^4^. In 1938, Hallpike and Cairns first showed that EH was closely related to Meniere’s disease in temporal bone specimens and may represent a pathological change of MD^5^. Later studies successfully discovered that most patients with MD had EH, and the proportion of EH was significantly higher in patients with MD than in those with other ear diseases and in the healthy population^6^, ^7^. Basic research established EH animal models through embolization, immunization, and hormones^8^, ^9^. Desmopressin acetate (Desmopressin, DDAVP) is a vasopressin analog and a V2 receptor agonist that induces EH in animal models^10^, while the V2 receptor antagonist, OPC-41061, antagonizes DDAVP stimulation and reduces EH level in guinea pigs^11^. In this study, we intraperitoneally injected DDAVP to induce EH in guinea pigs.

With the discovery of the second messenger Cyclic Adenosine Monophosphate (cAMP) by Sutherland in 1957^12^, cAMP has been shown to mediate the intracellular functions of epinephrine and glucagon, ushering in an era of signal transduction research. The three main targets of cAMP are Protein Kkinase A (PKA, A cell signal transduction factor)^13^, Exchange Proteins Directly Activated by cAMP (Epacs)^14^, ^15^, and cyclic nucleotide-gated channels^16^, which regulate various cellular activities. In 1968, cAMP was reported to be the only PKA and effector molecule^17^. However, 30 years later, two independent research groups showed a new cAMP signaling pathway. In 1998, Graybiel et al. discovered a new cAMP signaling pathway family, termed cAMP-regulated guanine nucleotide exchange factors I and II (cAMP-GEFI and II), corresponding to Epac1 and Epac2, respectively, which overturned the traditional understanding^15^. Simultaneously, in 1998, de Rooij and Bos conducted a sequence homology search with the cAMP-binding domain sequence of the PKA R subunit, which revealed that Epac1, as a novel cAMP signaling pathway, mediated PKA-independent Rap1 activation in response to Camp^14^. Since then, Epac1 has been extensively studied and reported to be involved in the regulation of various cellular functions, such as participating in intracellular and extracellular signal transmission, enhancing cell adhesion, and regulating vascular endothelial cell barrier, cell proliferation, and apoptosis^18^, ^19^.

Relevant literature shows that cAMP, as an intracellular second messenger, could be stimulated by vasopressin to activate PKA^20^ and then dynamically regulate the water and electrolyte metabolism of the inner ear by modulating the expression of Aquaporins (AQPs), many potassium channel proteins, and Na^+^-K^+^-ATPase in the stria vascularis and endolymphatic sac, thereby altering the hearing and vestibular function. Activated by DDAVP, cAMP-PKA, upregulates AQP2 expression on the luminal side of collecting duct epithelial cells to increase water reabsorption and concentrate urine. However, the application of H89 (Isoquinoline sulfonamides, Protein Kinase Inhibitors) alone, the PKA inhibitor, could only partially inhibits DDAVP-induced urinary concentration; while combined application of H89 and 8-pCPT, Epac-specific agonist, could noticeably upregulates AQP2 expression and enhances urinary concentration^21^, ^22^, indicating that the cAMP-Epac1 signaling pathway plays an important role in the regulation of renal water and salt metabolism. Nevertheless, no previous study has examined whether cAMP-Epac1 also plays an important role in the DDAVP-mediated regulation of AQP2 and induction of EH in the inner ear. In addition, our previous study showed that both Epac1 and Epac2 mRNAs and proteins were expressed in the inner ear of guinea pigs, as well as uniformly expressed in hair cells, the spiral ganglia, basilar membrane, saccules, and utricles. The expression was more obvious in capillary endothelial cells in the stria vascularis, suggesting that the cAMP-Epac1 signaling pathway may play an important role in maintaining the normal physiological function of the blood-labyrinth barrier and regulating the stability of the inner ear microcirculation^23^. Hence, in this study, we sought to explore whether cAMP-Epac1 signaling is activated during DDAVP-induced EH, with the aim to provide new insight for further in-depth study of DDAVP-induced EH.

## Methods

### Ethical approval

This study was approved by the Laboratory Animal Ethics Committee of Xuzhou Medical University, Jiangsu Province, China (approval number: L20210226392). All animal experiments in this study were conducted in accordance with ethical requirements.

### Animals

Eighteen healthy, red-eyed guinea pigs (36 ears) weighing 200–350 g were randomly divided into three groups (6 guinea pigs and 12 ears per group), as follows: The control group, which received intraperitoneal injection of sterile saline (same volume as that in the other two groups) for 7 consecutive days; the DDAVP-7d group, which received intraperitoneal injection of 10 μg/mL/kg DDAVP for 7 consecutive days; and the DDAVP-14d group, which received intraperitoneal injection of 10 μg/mL/kg DDAVP for 14 consecutive days. The three groups of guinea pigs were subjected to the same sample collection methods by injecting 3% pentobarbital and euthanizing via decapitation. The bilateral temporal bones of each animal were quickly removed, followed by opening the otic vesicle and removing the excess bone. The excess bone of the 12 cochleae in each group were immediately removed under the microscope; six of which were stored in RNAlater solution at −20 °C for Reverse Transcription (RT)-PCR, and the other six were stored at −80 °C for western blotting.

### Analysis of Epac1, Epac2, and Rap1 mRNA expression by RT-PCR

RT-PCR was used to determine the mRNA expression of Epac1, Epac2, and Rap1 using the specific primers designed by Primer 6 software (Table 1).

**Table 1:** Primer 6 software was used to design the primers in the present study.

Gene	Forward	Reverse
Epac1	AGCCACCATCATCCTGCGAGAA	CACCACCTTGCCGTGTTCTTCT
Epac2	CGAGTCATCCGCCTGGTTCTTC	TTCTGGCAGTTGCTCCTTGAGG
Rap1A	TTCAGGAGGCGTGGGAAAGTC	TCCAGCATACACTGTTGGCAAT
Rap1B	GCTAGTCGTTCTTGGCTCAGGA	ACACTGCTGTGCATCTACTTCA
Rap1gap	ACGTGGCTGGAGGACAGTGT	GAAGTGTGCGGCTCAGATGCT
GAPDH	AAGGCTGTGGGCAAGGTCATCC	TTCTCCAGGCGGCAGGTCAGAT

The total RNA samples were extracted from the cochleae of individual groups according to the manufacturer’s instructions, followed by determining the RNA concentration and quality and performing reverse transcription for cDNA synthesis. The reaction system for RT-PCR included 10 μL of 2× RT-PCR Master Mix (SYBR Green), 1 μL template (10-fold dilution of cDNA), 2 μL of primer MIX (10 μM of forward/reverse primers), and 7 μL of 0.1% DEPC water, to a total volume of 20 μL. RT-PCR was performed in a PCR system for amplification and preparation of the dissolution curve of each target gene. The Cycle threshold (Ct) value was derived and recorded to calculate the relative mRNA expression of individual target genes using the 2^−ΔΔ^Ct value.

### Analysis of Epac1, Epac2, and Rap1 protein expression by western blotting

Western blotting was used to detect the protein expression of Epac1, Epac2, and Rap1 in the cochleae of guinea pigs in each group. As described above, sampling of cochleae only preserves the lateral wall of each cochlea. Six cochleae from each group of animals were used for total protein extraction and western blotting.

In brief, the lateral wall tissue of the cochlea was cut into small pieces and mixed with a triple pack of enzyme inhibitors within 10 min before use. Subsequently, the tissues were dissolved and mixed in RIPA lysis buffer (100 µL lysis buffer for every 20-mg tissue), before homogenizing the tissue by repeatedly grinding until no visible tissue chunks remained in the lysate to ensure sufficient lysis.

The total protein extraction was performed using a commercial protein extraction kit according to the manufacturer’s instructions (KGP250, Jiangsu KeyGen Biotech, Jiangsu Province, China) and was quantified by a bicinchoninic acid assay (KGA902, BCA protein content detection kit, Jiangsu KeyGen Biotech.). The Gel-Pro32 software package was used to analyze the scale of the target protein bands on the blots.

The primary and secondary antibodies used for western blotting were as follows: rabbit anti-GAPDH primary antibody (KGAA002, 37 kDa, 1:5,000), Horseradish Peroxidase (HRP)-conjugated goat anti-mouse IgG secondary antibody (KGAA37), and HRP-conjugated goat anti-rabbit IgG secondary antibody (KGAA35) (all Jiangsu KeyGen Biotech), rabbit anti-Epac1 primary antibody (GTX41235, Gene Tex, 104 kDa, 1:500), mouse anti-Epac2 primary antibody (SC-28326, Santa Cruz Biotechnology, Dallas, TX, 126 kDa, 1:200), and mouse anti-Rap1 primary antibody (SC-53434, Santa Cruz Biotechnology, 44 kDa, 1:200).

### Statistical analysis

The SPSS 20.0 software package (IBM, Armonk, NY) was used to conduct all statistical analysis. Normally distributed data were analyzed by independent sample *t*-test and one-way analysis of variance. Non-normally distributed data were compared using two independent sample nonparametric analysis (Mann-Whitney test) between two groups, and multiple independent sample nonparametric analysis (Kruskal-Wallis test) between multiple groups. The level of significance was set as α = 0.05; *p*-values < 0.05 were considered to indicate a significant difference.

## Results

### Relative mRNA expression of Epac1, Epac2, Rap1A, Rap1B, and Rap1gap detected by RT-PCR

**Figure 1:**
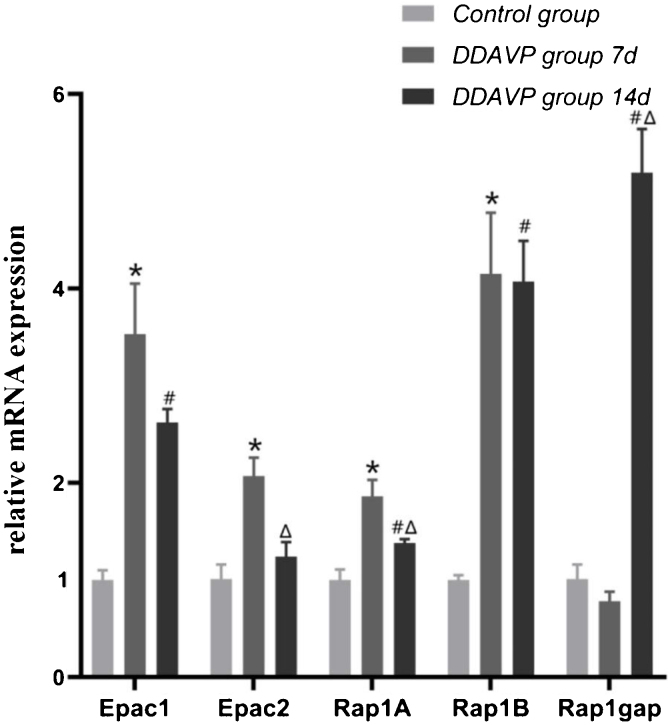
Relative mRNA expression of Epac1, Epac2, Rap1A, Rap1B, and Rap1gap in the lateral wall of the cochleae of the control and DDAVP treatment groups.

Fig. 1 and Table 2 show the relative mRNA expression of Epac1, Epac2, Rap1A, Rap1B, and Rap1gap in the guinea pig cochleae among the three groups.

1) Compared to the control group, the relative mRNA expression of Epac1, Epac2, Rap1A, and Rap1B in the cochlea tissue of the DDAVP-7d group was significantly higher (*p* <  0.05), while no significant difference in Rap1 GTPase activating protein (Rap1gap) mRNA expression was found between the two groups (*p* =  0.127).
2) The relative mRNA expression of Epac1, Rap1A, Rap1B, and Rap1gap in the cochlea tissue of the DDAVP-14d group was significantly higher than that of the control group (*p* <  0.05), while no significant difference in Epac2 mRNA expression was found between the DDAVP-14d and control groups (*p* =  0.133).
3) Comparison between the DDAVP-14d and DDAVP-7d groups showed that the DDAVP-14d group had significantly lower Epac2 and Rap1A (*p* <  0.05) and higher Rap1gap (*p* <  0.05) mRNA expression than that of the DDAVP-7d group, whereas no significant differences in Epac1 and Rap1B mRNA expression were found between the two groups (*p* =  0.074 for Epac1 and *p* = 0.127 for Rap1B).

**Table 2:** The relative mRNA expression of Epac1, Epac2, Rap1A, Rap1B and, Rap1gap.

	Control group	DDAVP-7d group	DDAVP-14d group
Epac1	1 ± 0.11	3.53 ± 0.52^a^	2.62 ± 0.41^b^
Epac2	1.01 ± 0.15	2.07 ± 0.19^a^	1.24 ± 0.15^c^
Rap1A	1 ± 0.11	1.86 ± 0.17^a^	1.38 ± 0.04^b^, ^c^
Rap1B	1 ± 0.05	4.15 ± 0.63^a^	4.07 ± 0.42^b^
Rap1gap	1.01 ± 0.15	0.78 ± 0.1	5.19 ± 0.45^b^, ^c^

a Control group vs. DDAVP-7d group, *p* <  0.05.

b Control group vs. DDAVP-14d group, *p* <  0.05.

c DDAVP-7d group vs. DDAVP-14d group, *p* <  0.05.

### Protein expression of Epac1, Epac2, and Rap1 detected by western blotting

**Figure 2:**
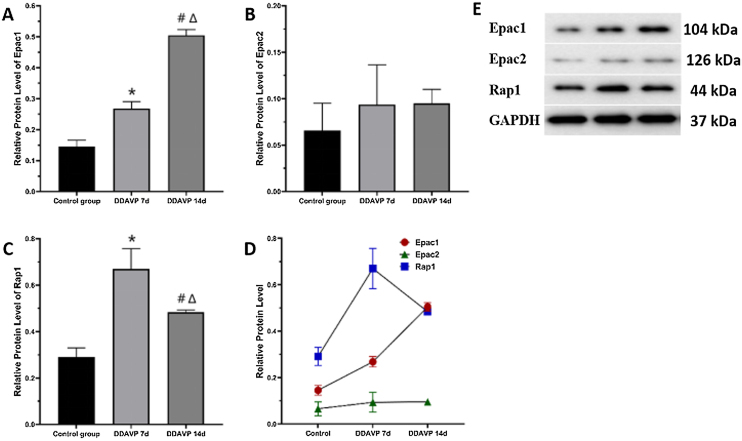
Epac1, Epac2, and Rap1 protein expression in the cochleae of different groups.

Fig. 2 and Table 3 show the relative protein expression of Epac1, Epac2, and Rap1 in the guinea pig cochleae among the three groups.

1) The Epac1 protein expression in the cochlea tissue was the highest in the DDAVP-14d group, followed by that in the DDAVP-7d group, and was the lowest in the control group, showing significant differences between the groups (*p* <  0.05).
2) No significant differences in Epac2 protein expression in the cochlea tissue were found among the three groups (control group vs. DDAVP-7d group, *p* =  0.513; control group vs. DDAVP-14d group, *p* =  0.127; DDAVP-7d group vs. DDAVP-14d group, *p* =  0.513).
3) Rap1 protein expression in the cochlea tissue was the highest in the DDAVP-7d group, followed by that in the DDAVP-14d group, and was the lowest in the control group, showing significant differences between the groups (*p* <  0.05).

**Table 3:** The relative protein expression of Epac1, Epac2, Rap1.

	Control group	DDAVP-7d group	DDAVP-14d group
Epac1	0.15 ± 0.02	0.27 ± 0.02^a^	0.50 ± 0.02^b^, ^c^
Epac2	0.07 ± 0.03	0.09 ± 0.04	0.10 ± 0.02
Rap1	0.29 ± 0.04	0.67 ± 0.09^a^	0.48 ± 0.01^b^, ^c^

a Control group vs. DDAVP-7d group, *p* <  0.05.

b Control group vs. DDAVP-14d group, *p* <  0.05.

c DDAVP-7d group vs. DDAVP-14d group, *p* < 0.05.

## Discussion

EH is mainly caused by excessive production or reduced absorption of endolymph, which is mainly a highly potassium hypertonic fluid, whereas perilymph is an isotonic fluid containing a large number of sodium ions. The Na^+^-K^+^-ATPases of the reissner's membrane, stria vascularis, and endolymphatic sac continuously transport K^+^ to the endolymph, thereby maintaining the dynamic balance of the endolymph. Additionally, AQPs present in the apical membrane of the endolymphatic sac^24^, ^25^ could be regulated by Arginine Vasopressin (AVP) to maintain the electrolyte balance of the inner ear. Known as an antidiuretic hormone, AVP exerts its effect through V2 receptor in the body. Also as a V2-specific receptor agonist, DDAVP plays an important role in maintaining the water and salt metabolism in the body^26^. Takeda^9^ established a model of endolymphatic hydrops by injecting arginine vasopressin into the abdomen of guinea pigs. Katagir^27^ found that prolongation of the injection of arginine vasopressin increased the degree of hydrops in the animal membrane labyrinthia, with a 100 percent success rate. We constructed EH guinea Pigs model through intraperitoneal injection of DDAVP, which is commonly used in this field, to further explore the changes of Epac1 in the DDAVP-induced EH model.

More than a decade after the discovery of cAMP, PKA was the second protein kinase to be discovered after phosphorylated kinases^28^ and has been extensively studied in the following years. However, with continued research progress, studies have shown that not all the transduction effects of cAMP are mediated by PKA, which has led many scientists to speculate that the PKA-independent cAMP signaling pathways may be involved in mediating cell signaling. Different from PKA proteins, which are composed of separated R and C subunits encoded by different genes, Epac protein, as single polypeptide molecules, has also been studied in this field^14^, ^15^. The NH_2_-terminal regulatory region of Epac may be derived from the R submit of PKA, while the COOH-terminal catalytic region of Epac is closely related to Ras superfamily proteins^29^. Epac has two subtypes, including Epac1 and Epac2; Epac1 is mainly expressed in the kidney, breast, skeletal muscle, thyroid, and brain, whereas Epac2 is mainly associated with the central nervous system and adrenal gland. We previously showed that Epac1 mRNA is extensively expressed in the inner ear, heart, liver, kidneys, intestines, and lungs^23^. Among those organs, Epac1 mRNA was highly expressed in the liver, kidneys, and intestines, whereas Epac2 mRNA was expressed in the inner ear and heart but not in the liver, kidneys, intestines, and lungs. These results were consistent with the previous literature.

DDAVP could upregulate the expression of AQP2 by activating the cAMP-PKA signaling pathway^30^. However, the expression of AQP2 was only partially reduced by the PKA inhibitors H89 and KT-5720 (PKA Inhibitor VII). In contrast, simultaneous administration of H89 and Epac selective agonists still upregulated the expression of AQP2^21^, ^22^, indicating that cAMP-Epac signaling pathway also essentially involved in maintaining hydroelectrolyte balance by regulating the expression of AQP2 in the kidney. Epac either acts synergistically with PKA or independently by activating the downstream signaling molecule, small G protein Rap1^14^. Rap1 is divided into two subtypes: Rap1A and Rap1B, which are key signaling molecules in the regulation of vascular endothelial stability^31^. Rap1gap enhances GTPase activity, leading to GTP hydrolysis, thereby inactivating Rap1^32^. In this study, the Rap1gap mRNA expression in the cochleae of guinea pigs of the DDAVP-14d group was significantly higher than that in the DDAVP-7d group and control group. We speculated that the increase in Rap1gap mRNA expression of the guinea pigs with longer DDAVP injection was related to the cAMP-Epac1 signaling pathway by stimulating the downstream signaling factor Rap1, increasing Rap1gap activity, whereas the compensatory increase in Rap1Gap inactivated part of Rap1 to maintain homeostasis. In the presence of Rap1B, disrupting the signal transduction of Rap1A does not affect the functions of endothelial cells^33^. Therefore, Rap1B plays a major role in maintaining the function of vascular endothelial cells. The RT-PCR results also showed that Rap1B mRNA expression was significantly higher than that of Rap1A in the DDAVP-treatment groups, which was consistent with the findings of previous studies. We speculate that Rap1B may play an important role in maintaining endothelial function of the guinea pig inner ear.

The Epac1-Rap1 signaling pathway may play a direct role in thrombin-induced barrier disruption^34^. Administration of an Epac-selective agonist could induce a Rap1-dependent increase in cortactin, thereby enhancing endothelial barrier function. This evidence indicates that the cAMP-Epac1 signaling pathway underlies the permeability and barrier function of vascular endothelial cells *in vivo*. In our previous study^23^, ({Wang, 2022 #40}} both mRNA and proteins of Epac1 and Epac2 were expressed in the inner ear of guinea pigs, which were uniformly expressed in the hair cells, the spiral ganglia, basilar membrane, saccules, and utricles, while the expression was more obvious and characteristic in capillary endothelial cells in the stria vascularis, implying that cAMP-Epac1 signaling pathway may be essentially involved in maintaining the function of the blood-labyrinth barrier and regulating the stability of microcirculation in the inner ear. In this study, compared to the control group, the Epac1, Epac2, and Rap1 mRNA expression was significantly increased after intraperitoneal injection of DDAVP for 7 and 14 consecutive days, and the Epac1 mRNA expression was significantly higher than the Epac2 mRNA expression. Relevant literature has shown that the expression of Epac1 and Epac2 is regulated by development. Indeed, Ishikawa et al. analyzed that Epac1 and Epac2 mRNA expression in the brain, heart, kidneys, and lungs of humans at different stages of development and showed that both genes were highly expressed, although expression levels decreased with growth and development^35^. In this study, Epac was activated after intraperitoneally injecting DDAVP, with the evidence that the expression of Epac1 and Epac2 mRNA in the DDAVP-7d and -14d group was significantly higher than that of the control group. According to a protein sequence analysis, the protein structures of Epac1 and Epac2 showed some similarities. For example, both proteins contain an NH2-terminal regulatory domain and a COOH-terminal catalytic domain^15^. Moreover, both regulatory domains of Epac1 and Epac2 contain a Dishevelled/EGL-10/Pleckstrin (DEP) domain and Cyclic Nucleotide Binding (CNB) domain, which present in PKA, PKG, and in ion channels regulated by the guanine nucleotide change factor Epac and cyclic nucleotides^36^. Unlike the Epac1 protein, the NH2-terminal regulatory region of Epac2 protein possesses an additional CNB-A domain, which has a lower affinity for cAMP. Our results showed, in both the DDAVP-7d and DDAVP-14d groups, the expression of Epac1 protein was significantly higher than that of Epac2 protein in the lateral wall of cochleae of guinea pigs the reason of which may be the affinity of Epac2 protein for cAMP was much lower than that of Epac1 protein.

Rehmann and Bos showed that Epac induced the binding of cAMP and Rap1, and the binding of cAMP to Epac1 was tighter in the presence of Rap1^37^. Similarly, in our results, the expression of Rap1 protein in the DDAVP-treatment groups was significantly higher than that in the control group. We speculate that in the presence of Rap1, the fact that Epac1 protein expression in the DDAVP-14d group did not decrease was likely because Epac1 bound tightly to Rap1, making its degradation difficult.

Åsrud et al. reported impaired urinary concentration effect of DDAVP in Epac1-deficient mice and significantly reduced expression of tight junction proteins in the renal collecting ducts, suggesting lessened effect of cAMP mediated by DDAVP without the presence of Epac1^38^. Based on these findings and our results, we speculate that, similar to the kidneys, cAMP-Epac1 was activated by DDAVP in the guinea pig inner ear and underlied the EH developing, during which process the tight junction proteins in the endothelial cells was upregulated to increase the barrier function and reduce the barrier permeability. These hypotheses are our next objectives to further confirm.

Despite its strengths, this study also has some limitations. First, we demonstrated activation of the cAMP-Epac1-Rap1 signaling pathway in the cochlea of the DDAVP-induced guinea pig EH model but did not reveal whether the activation was directly involved in DDAVP-induced EH or was only a side effect of DDAVP. In other words, it is still unclear if the activation of the cAMP-Epac-Rap1 signaling pathway is a causative factor for DDAVP-induced EH or just an epiphenomenon. In the follow-up study, we will further investigate the interaction between the activation of the cAMP-Epac1-Rap1 signaling pathway and the effect of DDAVP on EH development to establish new molecular targets for treating MD.

## Conclusion

This study showed for the first time that DDAVP upregulated the expression of Epac1 protein in guinea pig cochleae to further activate the cAMP-Epac1-Rap1 signaling pathway, which may be a mechanism of DDAVP-induced EH in guinea pigs.

## Fundings

The Development Projects for Science and Technology of Chinese Medicine of Jiangsu Province (QN202009); Key R & D Plan (Social Development) Project of Xuzhou Science and Technology Bureau (KC20067); 10.13039/501100001809National Natural Science Foundation of China (81900942, 52275296); Medical science and technology innovation project of Xuzhou Municipal Health Commission (XWKYHT20220149); Open project of key laboratories in colleges and universities in Jiangsu Province (XZSYSKF2022005).

## Conflicts of interest

The authors declare no conflicts of interest.
